# Impact of the distribution of recovery rates on disease spreading in complex networks

**DOI:** 10.1103/PhysRevResearch.2.013046

**Published:** 2020-01-14

**Authors:** Guilherme Ferraz de Arruda, Giovanni Petri, Francisco A. Rodrigues, Yamir Moreno

**Affiliations:** ^1^ ISI Foundation, Via Chisola 5, 10126 Torino, Italy; ^2^ Departamento de Matemática Aplicada e Estatística, Instituto de Ciências Matemáticas e de Computação, Universidade de São Paulo, Campus de São Carlos, Caixa Postal 668, 13560-970 São Carlos, São Paulo, Brazil; ^3^ Institute for Biocomputation and Physics of Complex Systems and Department of Theoretical Physics, University of Zaragoza, 50018 Zaragoza, Spain

## Abstract

We study a general epidemic model with arbitrary recovery rate distributions. This simple deviation from the standard setup is sufficient to prove that heterogeneity in the dynamical parameters can be as important as the more studied structural heterogeneity. Our analytical solution is able to predict the shift in the critical properties induced by heterogeneous recovery rates. We find that the critical value of infectivity tends to be smaller than the one predicted by quenched mean-field approaches in the homogeneous case and that it can be linked to the variance of the recovery rates. Our findings also illustrate the role of dynamical-structural correlations, where we allow a power-law network to dynamically behave as a homogeneous structure by an appropriate tuning of its recovery rates. Overall, our results demonstrate that heterogeneity in the recovery rates, eventually in all dynamical parameters, is as important as the structural heterogeneity.

## I. INTRODUCTION

Heterogeneity, whether in the nature of the components or the pattern of connections, is a key characteristic of complex systems. This is particularly evident in the case of the spreading of a disease in a networked population, where the inclusion of structural heterogeneity has long been known to radically change the process's critical behavior [1–6]. As an illustration, consider two classical contagion models, the susceptible-infected-susceptible (SIS) and the susceptible-infected-recovered (SIR) models. On a homogeneous network, they both present a nonvanishing critical point [5,6]. However, the introduction of structural heterogeneity, in the form of broad degree distributions of the nodes, can result in a vanishing critical point [1,5–7]. More specifically, in the thermodynamic limit, a divergence of the second moment of the degree distribution [1,5,6,8] or a divergence in the maximum degree [1,5,6,8] implies a vanishing critical infectivity. This in turn has important practical implications for real-world networks, because many of them display very broad [7–9] (or even scale-free) degree distributions [10]. References [11–18] considered, in contrast, homogeneous structures but accounted for arbitrary times in the state transitions. Interestingly, previous works studied models characterized by a series of infected states (without a biological interpretation) that described the global behavior accurately [12–15,17]. Notably, the model proposed in [12] is general, but each transition has an assigned function, which complicates the model, making its practical use limited. These works encounter, however, a fundamental limitation, because they describe the population at the mean-field level [11–18]. While structural and dynamical heterogeneities are independently accounted for, modeling both types of heterogeneities together has received considerably less attention until recently. Indeed, it was mainly studied for the SIR model: A message-passing formalism was proposed in [19,20] and a heterogeneous mean-field approach in [21]. In the latter, the authors also performed numerical experiments showing that the population can be more vulnerable in the scenario with dynamical heterogeneity. More recently, this problem was investigated on temporal networks [22] using an SIS process, which was described within the quenched mean-field (QMF) formalism and mainly focusing on spreading rates [23,24]. A similar approach was considered in [25], where however the authors highlighted a different aspect, i.e., the allocation of resources during an outbreak.

Here we investigate a different type of dynamical heterogeneity by characterizing the critical properties of an SIS model when recovery rates are distributed heterogeneously across the population. Heterogeneous recovery rate distributions can be associated with biological differences between individuals [26,27], demographic characteristics [28], and social differences that result in nonhomogeneous access to the health system [29]. Additionally, we also consider the case in which correlations arise between the structure and dynamics. We show, both analytically and numerically, that such correlations can induce opposing and unexpected dynamical outcomes, for example, power-law (PL) networks displaying nonvanishing critical points and conversely homogeneous networks displaying vanishing critical points. Indeed, we show that the standard QMF predictions, which are a lower bound in the standard scenario, no longer provide such a bound in the heterogeneous rate case. We then propose a simple formulation to overcome this and provide a different lower bound for the process. Our results complement previous evidence on the SIR model [21] and imply that proper characterization of the dynamical parameters is of utmost importance not only for a better understanding of spreading processes, but also for many practical applications, such as surveillance, forecasting, resource management, and network reconstruction, among many others.

## II. THE SIS MODEL WITH HETEROGENEOUS RECOVERIES

We start by considering a population composed of N individuals with an arbitrary pattern of connections in a single connected component. These connections can be represented as a network and are described by the network's (usually symmetric) adjacency matrix A. Each individual can be in one of two states: (i) infected ($Y_{i}=1$) or (ii) susceptible ($X_{i}=1$). Using a Markovian approach, the epidemic process is modeled as a collection of independent Poisson processes. In order to model the spreading of the disease through the network of contacts, with each directed edge i∼j, emanating from the infected individual i, we associate a Poisson process with rate $\lambda_{ij}$, $N^{\lambda_{ij}}\left(t\right)$ (whose transitions are $Y_{i}+X_{j}\to Y_{i}+Y_{j}$). Additionally, with each infected individual, we associate a Poisson process with rate $\delta_{i}$, $N^{\delta_{i}}\left(t\right)$, modeling the recovery ($Y_{i}\to X_{i}$). This system is statistically described using the order parameter ρ and the susceptibility χ, defined as

(1) $$\rho =\frac{1}{N}\sum_{i}^{N}\langle Y_{i}\rangle ,\;\chi =\frac{\langle {n_{I}}^{2}\rangle -{\langle n_{I}\rangle }^{2}}{\langle n_{I}\rangle },$$

where $n_{I}$ is the number of infected individuals. Both quantities can be directly estimated using Monte Carlo methods, in particular, the quasistationary method and the Gillespie algorithm, where each of the processes mentioned above is simulated, and the state of the nodes is evaluated [6].

In the QMF approach, one implicitly assumes that $\langle X_{i}Y_{j}\rangle \approx \langle X_{i}\rangle \langle Y_{j}\rangle$. Physically, this corresponds to neglecting dynamical correlations. Defining $y_{i}=\langle Y_{i}\rangle$, the process is described as

(2) $$\frac{dy_{i}}{dt}=-\delta_{i}y_{i}+\left(1-y_{i}\right)\sum_{j}\lambda_{ij}A_{ij}y_{j}.$$

Thus, defining $\Delta_{ii}=\delta_{i}$, $W=\lambda_{ij}$, and $Q=\Delta^{-1}\left(A\circ W\right)$, the critical point is given as

(3) $$\lambda_{c}^{\mathrm{QMF}}={\left[\Lambda_{\text{max}}\left(Q\right)\right]}^{-1},$$

where $\Lambda_{\text{max}}\left(Q\right)$ is the leading eigenvalue of Q. Note that the elements of Q are the expected number of contacts before recovery. Obviously, the critical point simplifies to $\tau_{c}^{\mathrm{QMF},\mathrm{std}}={\left(\frac{\lambda }{\delta }\right)}_{c}={\left[\Lambda_{\text{max}}\left(A\right)\right]}^{-1}$ in the homogeneous case, i.e., when $\delta_{i}=\delta$ and $\lambda_{ij}=\lambda$. Thus, in the thermodynamic limit, the critical point for PL networks goes to zero if the maximum degree is a growing function of the network size [5,6,8]. On the other hand, we can consider a scenario that is equivalent to the contact process (CP) by setting the spreading rates as $\lambda_{ij}=\frac{\lambda }{k_{i}}$, which is thus described by the probability transition matrix $P_{ij}=\frac{A_{ij}}{k_{i}}$. In this case, the critical point is finite and $\tau_{c}^{\mathrm{QMF},\mathrm{CP}}=1$, regardless of the underlying structure. Note that, similarly to the homogeneous case, this prediction [Eq. (3)] is an upper bound for the heterogeneous recovery rate scenario, because it relies on the independence of the random variables: If i∼j, then $P\left(Y_{i}=1|Y_{j}=1\right)\geq P\left(Y_{i}=1\right)=y_{i}$, which implies that the nodal probability is always overestimated (see [2] for a similar argument). From here onward, we set $\lambda_{ij}=\lambda$ and focus on the effect of the recovery rate distribution on the critical point. Considering an undirected network, from the matrix norm we can bound Eq. (3) using the standard QMF predictions as

(4) $$\min\left(\delta_{i}\right)\tau_{c}^{\mathrm{QMF},\mathrm{std}}\leq \lambda_{c}^{\mathrm{QMF}}\leq \max\left(\delta_{i}\right)\tau_{c}^{\mathrm{QMF},\mathrm{std}}.$$

We can see that Eq. (4) suggests that the standard QMF predictions might not be a lower bound to the alternative process. Note that the uncertainty assuming the standard QMF prediction increases as the variance also increases.

## III. SYNTHETIC NETWORKS

**FIG. 1.**
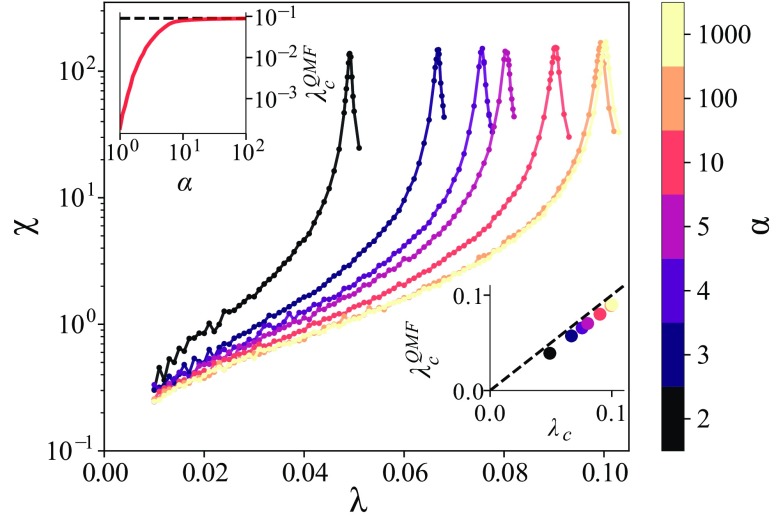
Susceptibility curves extracted from the Monte Carlo simulations for an Erdős-Rényi network with $N=10^{5}$ and 〈k〉≈10 considering that the recovery rate distribution follows an inverse-gamma distribution, whose shape parameter α is color coded. The insets show the dependence of $\lambda_{c}^{\mathrm{QMF}}$ on α (top) and a comparison with numerical results (bottom), also for different α.

To further characterize the critical behavior of our model, we first consider an Erdős-Rényi (ER) network with $N=10^{5}$ and 〈k〉≈10 (therefore $\tau_{c}^{\mathrm{QMF},\mathrm{std}}\approx 0.1$). This graph has a homogeneous structure and allows us to analyze the structural and dynamical effects independently. Mounting evidence shows that infectious times in real epidemics follow a gamma distribution [17,30,31]. Consequently, the rate distribution must follow an inverse-gamma distribution. We impose therefore the recovery rates to have an inverse-gamma distribution $\delta ∼\Gamma^{-1}\left(\alpha ,\beta \right)$, where α and β are the shape and scale parameters, respectively. Its mean is $\langle \delta_{i}\rangle =\frac{\beta }{\alpha -1}$ and its variance is $\text{Var}\left(\delta_{i}\right)=\frac{\beta^{2}}{{\left(\alpha -1\right)}^{2}\left(\alpha -2\right)}$ for α>2. To facilitate the comparison between different distributions, we restrict the distributions to unitary mean. In Fig. 1 we present the critical behavior of an ER network for different shapes α. The top inset emphasizes the behavior of the predicted critical point as a function of α and its comparison with estimations from Monte Carlo simulations. As expected, for sufficiently large values of α, the dynamics behave similarly to the standard SIS model with uniform δ, where the predicted threshold coincides (dashed line in the top inset). The agreement between analytical and simulated critical points is excellent, as it can be seen in the bottom inset of Fig. 1. Furthermore, as α decreases, the variance of δ and consequently the uncertainty of the bounds are also enlarged. However, we observed that the critical point systematically moved towards zero in our simulations, which is also consistent with Eq. (4). Note that the inverse-gamma distribution is asymmetric with respect to its average and centered at $\delta_{i}\leq \langle \delta_{i}\rangle$. This implies that the bounds in Eq. (4) are also asymmetric. Since the recovery rates are sampled from this distribution, we expect that the number of individuals that take longer to recover is greater than the number of individuals that recover fast. This suggests that, via infection/reinfection mechanisms, the disease can survive for lower values of λ as compared to the standard QMF predictions. In addition to the critical properties, we also show a different supercritical behavior; for example, the low-α regime of a network can be similar to, and even in some regions it can be mistaken for, the high-α regime on top of a network with a different structure. This multiplicity of supercritical behaviors also raises questions in network reconstruction models based on disease dynamics.

## IV. REAL-WORLD NETWORKS

**FIG. 2.**
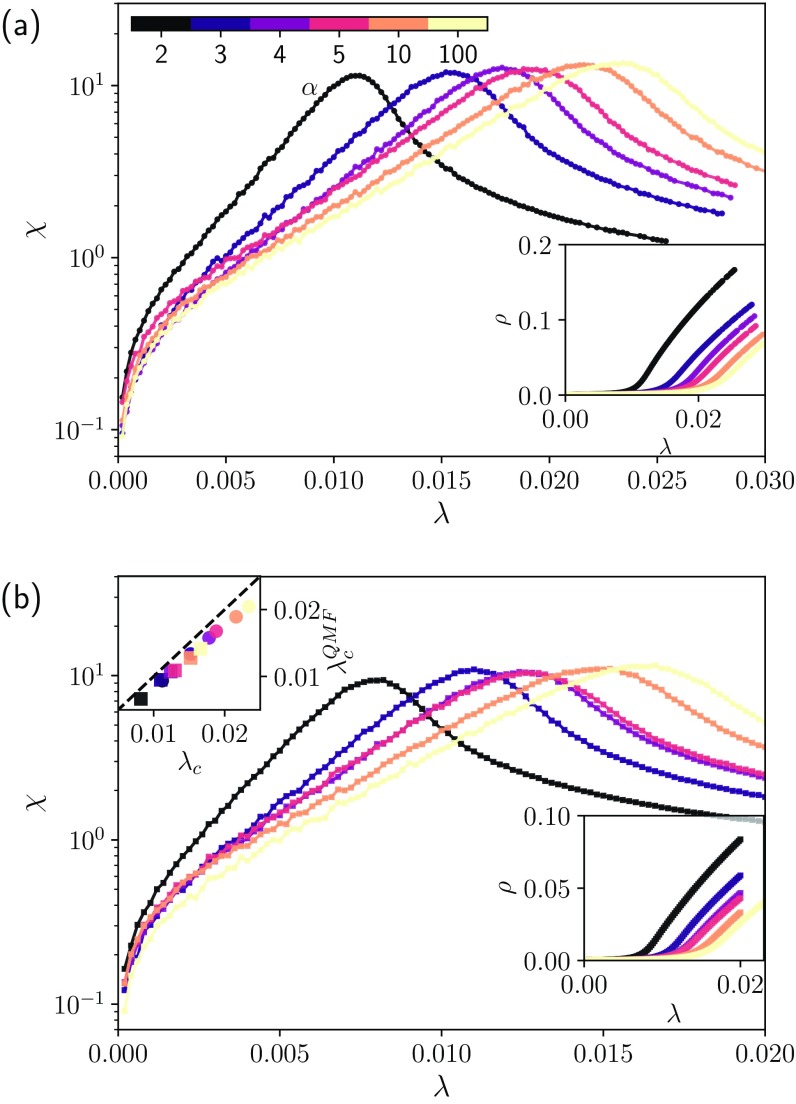
Results for the susceptibility when the dynamics are on top of real networks. We use (a) the UC Irvine messages social network [32,33] (•) and (b) the OpenFlights network [33,34] (▪). In both cases, we considered the undirected version of the giant component. In the main figure of each panel, we present the susceptibility for different values of α and λ. In the bottom right insets, we present the order parameter. In the top inset of (b), we present a comparison between the QMF estimated and predicted critical points.

We confirmed that our results hold in real-world networks. As before, we consider an inverse-gamma distribution. Figure 2 shows results of simulations in two real networks: the UC Irvine messages social network [Fig. 2(a)] [32,33] and the OpenFlights network [Fig. 2(b)] [33,34]. These networks represent different spatial scales of a similar spreading process: The social network corresponds to smaller scales and spatially localized systems, while the OpenFlights network captures a wider spatial scale. In the top inset of Fig. 2(b) we show that the critical point predictions are remarkably good for inverse-gamma recovery rates, even for these real networks.

Figures 1 and 2 reveal that the critical point decreases as we increase the variance of the recovery rate distribution. Thus, if we consider heterogeneous networks, assuming an average recovery rate in the QMF is not enough to provide an adequate characterization of the process. It is not a lower bound anymore, as can be seen in Fig. 1, where the numerically estimated critical point is always slightly lower than the value predicted by the standard QMF predictions ($\tau_{c}^{\mathrm{QMF},\mathrm{std}}\approx 0.1$). The proper correction for the QMF predictions is given by Eq. (3), which is a lower bound for the underlying process (see the bottom inset in Fig. 1).

## V. EFFECTS OF DYNAMICS-STRUCTURE CORRELATIONS

The bounds in Eq. (4) implicitly assume that there are no correlations between the structure and dynamics. From the Gershgorin circle theorem we know that every eigenvalue of Q lies at least in one of the disks $D(Q_{ii},R_{i})$ centered in $Q_{ii}$ with a radius given as $R_{i}=\sum_{i\neq j}\left|Q_{ij}\right|$. Therefore, considering a symmetric matrix, $|\Lambda_{k}|\leq Q_{ii}+R_{k}$, hence $\Lambda_{\max}\leq {∥Q∥}_{\infty }$, where the infinity norm is defined as

(5) $${∥Q∥}_{\infty }=\max_{1\leq i\leq N}\left(\sum_{j=1}^{N}\frac{A_{ij}}{\delta_{i}}\right)=\max_{1\leq i\leq N}\left(\frac{k_{i}}{\delta_{i}}\right).$$

If the structure and the dynamics are correlated, Eq. (5) might give us further insight. For instance, for the PL case, the leading eigenvalue of A diverges in the thermodynamic limit, leading to a vanishing critical point. Conversely, using Eq. (5) and a proper choice of $\delta_{i}$, we can change this behavior. In fact, assuming that $\delta_{i}\left(k_{i}\right)\propto k_{i}$ in the thermodynamic limit, we have

(6) $$\lim_{N\to \infty }{∥Q∥}_{\infty }=\lim_{N\to \infty }\left[\max_{1\leq i\leq N}\left(\frac{k_{i}}{\delta_{i}}\right)\right]=c,$$

where c<∞ is a finite real constant. This radically changes the critical behavior of the dynamics. Note that both the CP ($\lambda_{ij}=\frac{\lambda }{k_{i}}$) and the $\delta_{i}=k_{i}$ cases are described, at first order, by the probability transition matrix P, yielding $\tau_{c}^{\mathrm{QMF},\mathrm{CP}}=\lambda_{c}^{\mathrm{QMF}}=1$.

**FIG. 3.**
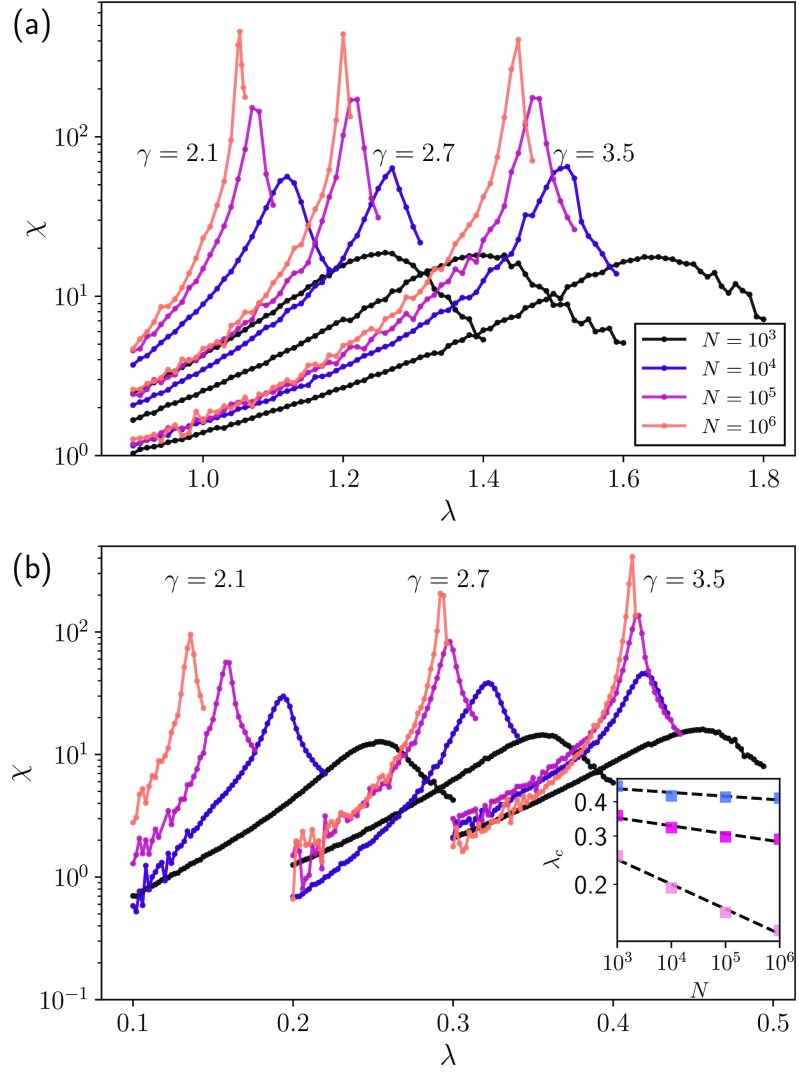
Finite-size analysis considering structure-dynamics correlations. We present the susceptibility (colors represent the sizes) in (a) the heterogeneous recovery rates, where $\delta_{i}=k_{i}$ on top of the power-law networks, whose exponents are γ=2.1,2.7,3.5, and (b) the SIS model with heterogeneous recovery rates, considering an Erdős-Rényi network and the recovery rates as $\delta_{i}=\frac{k_{i}}{k_{\text{PL}}}$, where $k_{\text{PL}}$ is a discrete power law, more specifically, the degree sequence of the networks used in (a). In the inset we present the critical point as a function of the system size in log-log scale.

In Fig. 3(a) we perform a finite-size analysis when the dynamics occurs on top of PL networks and recovery rates are $\delta_{i}=k_{i}$, finding evidence of a finite critical point, i.e., the value of λ corresponding to the peak of χ does not vanish. For comparison, the results for the CP are reported in Appendix E. We remark that the convergence towards the critical value for growing N seems to be slower in the CP case. In summary, our results show that a network with a power-law degree distribution may show a finite critical point for the SIS dynamics if degrees and recovery rates are appropriately correlated.

Next we show that the inverse scenario is also possible. Consider an ER network with 〈k〉≈10 and $\delta_{i}=\frac{k_{i}}{k_{\text{PL}}}$, where $k_{\text{PL}}$ is a discrete power law, i.e., $P\left(k_{\text{PL}}\right)∼k_{\text{PL}}^{-\gamma }$. That is, we now have a homogeneous structure and a heterogeneous recovery rate distribution. In Fig. 3(b) we show a finite-size analysis for this configuration with varying γ=2.1,2.7,3.5. We observe that for γ=2.7 and 3.5 the underlying structure plays an important role, suggesting a nonvanishing critical point or considerable reduction in the scaling exponent [see Fig. 3(b) inset, where both curves have a slope close to zero]. However, for γ=2.1 our results indicate the existence of a vanishing critical point [see Fig. 3(b) inset]. It seems reasonable to hypothesize that the scenario observed when γ=2.1 is due to the fact that, in the steady state, the infection probabilities are inversely proportional to the nodal recovery rates and thus that the evaluation of the recovery time at both ends of every edge enables an infection-reinfection mechanism. Understanding what the necessary and sufficient conditions to observe such a mechanism are and the interplay between structure and dynamics requires, however, further exploration.

In [8,35,36] it was shown that, depending on the network structure, the transition can be triggered by different activation mechanisms, namely, (i) collective (e.g., ER networks or the CP), (ii) k core (uncorrelated power law with 2<γ<2.5), or (iii) hub (uncorrelated power law with γ>2.5). Note that for uncorrelated power laws, this coincides with the different regimes of leading eigenvector localization [37] [for the analysis of the inverse participation ratio (IPR) of the leading eigenvector of Q we refer the reader to Appendix F]. Thus, we conjecture that it may be possible to alter these structural mechanisms with a proper recovery rate distribution, possibly considering dynamics-structure correlations. Note that we implicitly showed [Fig. 3(a)] how to transform k-core (γ=2.1) and hub-activated (γ=2.7,3.5) mechanisms into a collective behavior phenomenology. Importantly, our findings may lead to alternative prevention/intervention techniques that take advantage of the phenomenology reported here. We also highlight that the consequences of our findings are not limited to the cases explored here. For instance, it is natural to conjecture the existence of Griffith's phase in our setup. In this type of transition, we have an extended critical region instead of a single critical point, which was studied in complex networks in [36,38–42]. More specifically, in [39] the authors showed that slow dynamics on a weighed treelike structure can occur in a contact process. Its similarities to our scenario thus suggest that similar phenomenology is also possible in our case.

## VI. CONCLUSION

We have analyzed the impact of introducing heterogeneity in the recovery rates of an SIS disease dynamics. We showed that dynamical heterogeneity is as significant as structural heterogeneity and that it can induce drastic changes in the SIS critical properties. Furthermore, our results show that the standard QMF approach does not provide a lower bound for the heterogeneous case anymore. To solve this inconsistency, we proposed a solution that relates the structural and dynamical features via the spectral properties of a different matrix Q. This formulation presents opportunities for future research. For example, our findings raise questions about the consequences of heterogeneities in spreading rates and the interplay between spreading and recovery rates. Furthermore, with regard to control/containment strategies, heterogeneity in the recovery rates can be considered for intervention strategies (or even immunization, $\delta_{i}\to 0$). In contrast, heterogeneity of spreading rates would be related to prevention. In this context, our findings might also be of potential interest. In addition to the specific conclusions drawn here, there are others that concern more general aspects of disease spreading processes as well as the characterization of complex systems in general. For instance, differences in the localization properties induced by dynamical heterogeneities might influence the predictability of complex systems, and in particular of diseases [43], or in the reconstruction of networks from the dynamics as proposed in [44]. Finally, we also stress that our results might also have an impact on information spreading processes since individuals have different activity timescales, which could ultimately be related to the recovery time distribution.

## Figures and Tables

**FIG. 4.**
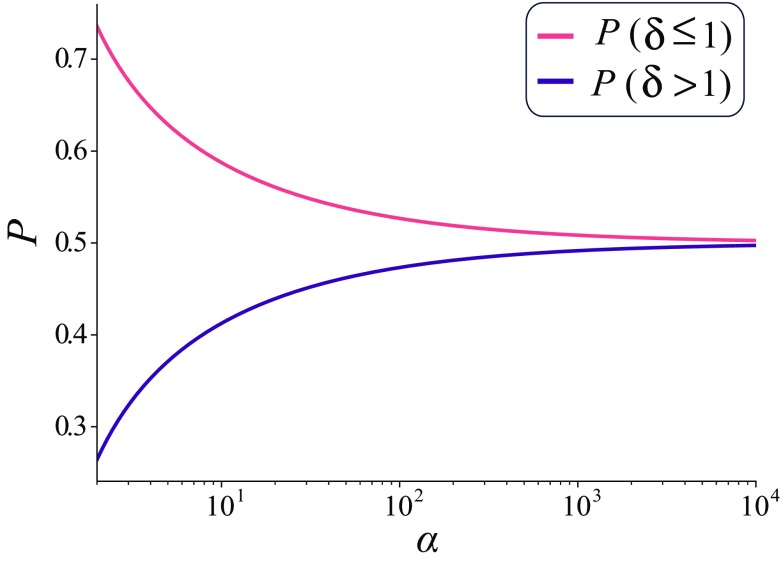
Evaluation of P(δ≤1) and P(δ>1) as a function of α, the shape parameter of an inverse-gamma distribution with β=α−1.

**FIG. 5.**
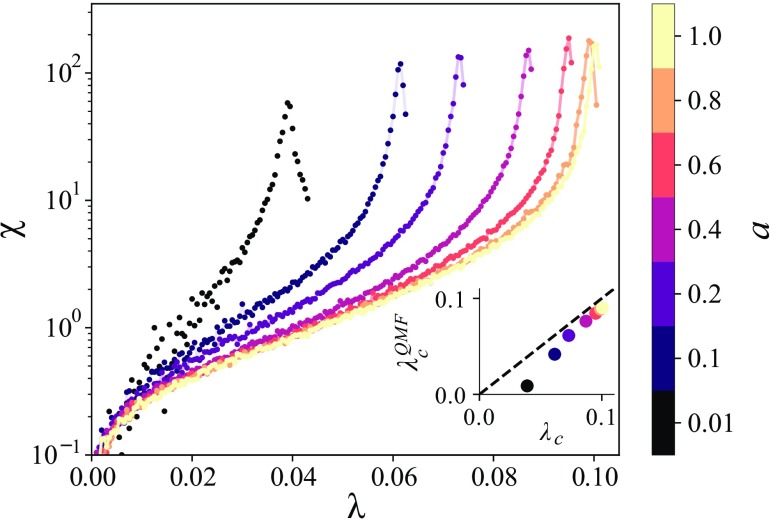
Susceptibility curves extracted from the Monte Carlo simulations for an Erdős-Rényi network with $N=10^{5}$ and 〈k〉≈10 considering that the rate distribution follows a uniform distribution (symmetric) U(a,2−a), where the parameter a controls the variance and is color coded.

**FIG. 6.**
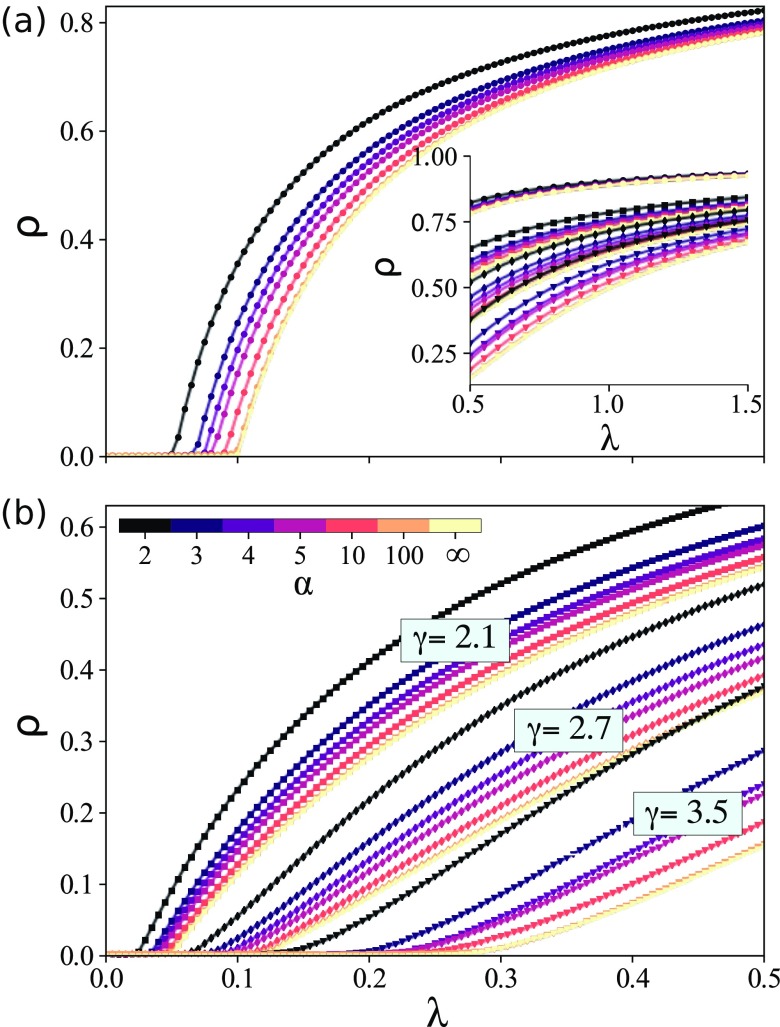
Phase diagram for (a) Erdős-Rényi (•) networks and (b) PL networks. In (b), from top to bottom, we have γ=2.1 (▪), γ=2.7 (⧫), and γ=3.5 (▾). In the inset in (a) we present the behavior for larger λ's in all the networks. They comprise $N=10^{4}$ nodes. The recovery rate distribution is given as an inverse-gamma distribution, whose shape parameter α is color coded.

**FIG. 7.**
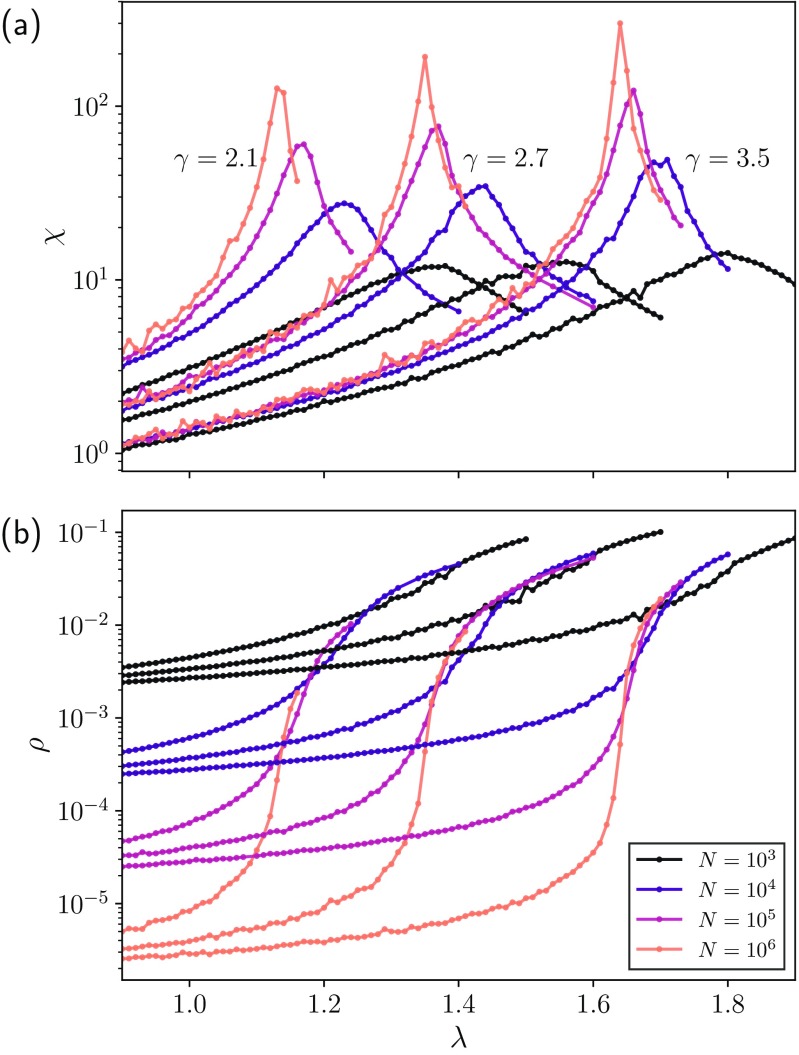
Finite-size analysis for the CP for (a) susceptibility and (b) the order parameter. The colors represent the sizes and from bottom to top, the curves are grouped by the power-law exponents γ=2.1,2.7,3.5, respectively.

**FIG. 8.**
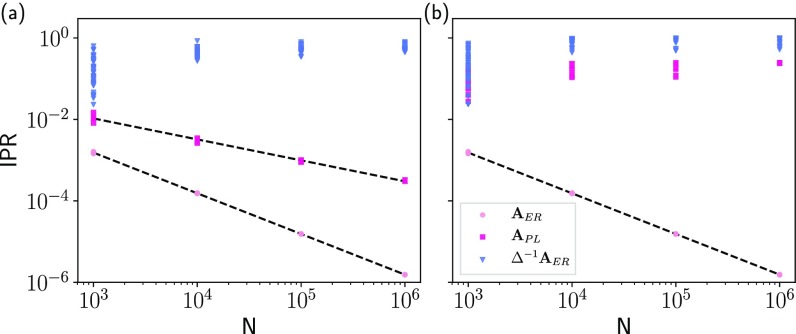
The IPR versus N for the Erdős-Rényi, PL networks and the correlated case $\Delta^{-1}A$, where $\Delta_{i}=\frac{k_{i}}{k_{\text{PL}}}$ for (a) γ=2.1 and (b) γ=3.5. Fifty networks were considered in each case.

## APPENDIX A: EQUATION (4) AS A BOUND FOR EQ. (3)

The critical point given by Eq. (3) can be bounded using a matrix norm. Thus, using the 2-norm, we obtain

(A1) $$\frac{{∥A∥}_{2}}{\Lambda_{\max}\left(\Delta \right)}\leq {∥\Delta^{-1}A∥}_{2}\leq \frac{{∥A∥}_{2}}{\Lambda_{\min}\left(\Delta \right)},$$

where ${∥A∥}_{2}=\sqrt{\Lambda_{\max}\left(A^{T}A\right)}$. Equation (A1) therefore provides bounds on the leading eigenvalue of Q given by the structure and the variance of $\delta_{i}$. Next, for undirected networks we have that ${∥A∥}_{2}=\Lambda_{\max}\left(A\right)={\left(\tau_{c}^{\mathrm{QMF}}\right)}^{-1}$. Moreover, the Perron-Frobenius theorem for non-negative matrices states that every non-negative matrix can be written as a limit of positive matrices, and therefore one has the existence of an eigenvector with non-negative components and the corresponding eigenvalue will be non-negative and greater than or equal (in absolute value) to all other eigenvalues [45]. Therefore, from the norm definition,

(A2) $${∥Q∥}_{2}=\sigma_{\max}\left(Q\right)=\max_{i}\left[\Lambda_{i}\left(Q\right)\right]$$

since Q is non-negative. Finally, inverting the equations and writing it in terms of the critical properties, we obtain Eq. (4).

## APPENDIX B: ANALYZING EQ. (4) FOR THE INVERSE-GAMMA DISTRIBUTION

The bounds obtained in Eq. (4) or (A1) cannot be further improved without knowledge about the relationship between the matrix A or W and $\Delta^{-1}$ [for more details about the effects of dynamics-structure correlations, see Eq. (5)]. Alternatively, one can use the asymmetries in the recovery rate distribution to understand how the lower and upper bounds behave. More specifically, the cumulative distribution for the inverse-gamma distribution is given as

(B1) $$P\left(\delta \leq x\right)=\frac{\Gamma \left(\alpha ,\frac{\beta }{x}\right)}{\Gamma (\alpha )}=Q\left(\alpha ,\frac{\beta }{x}\right),$$

where Γ(a,b) is the upper incomplete Gamma function, Γ(a) is the Gamma function, and Q(a,b) is the regularized gamma function. Next, keeping the average fixed, i.e., setting β=α−1, we can analyze how these bounds behave as a function of the shape α. We can calculate the probability that δ is larger or smaller than a give value x. Formally,

(B2) $$\begin{array}{ccc} P(\delta \leq x) & = & Q\left(\alpha ,\frac{\alpha -1}{x}\right), \end{array}$$

(B3) $$\begin{array}{ccc} P(\delta >x) & = & 1-Q\left(\alpha ,\frac{\alpha -1}{x}\right). \end{array}$$

Next, evaluating (B2) and (B3) for the average value $\langle \delta_{i}\rangle =x=1$, we obtain the fraction of individuals that have a recovery rate that is lower and higher, respectively, than average. Since $Q\left(\alpha ,\frac{\alpha -1}{x}\right)\geq \frac{1}{2}$ the inverse-gamma distribution is peaked below the average value, therefore suggesting that the lower bound decreases faster than the upper bound. Interestingly, α→∞ implies that $P\left(\delta \leq 1\right)=P\left(\delta >1\right)=\frac{1}{2}$, and thus the uncertainty is symmetrical. This is also depicted in Fig. 4.

We remark that the discussion of this Appendix concerns the uncertainty of the bounds and their asymmetry, which only suggests (but does not prove) that the critical point might decrease. This hypothesis is supported by our simulations.

## APPENDIX C: UNIFORM SYMMETRIC RECOVERY RATE DISTRIBUTION

In the main text we focused on the inverse-gamma distribution. This distribution is asymmetric. In this Appendix we focus on a symmetric scenario considering the uniform distribution U(a,2−a) whose average is $\langle \delta_{i}\rangle =1$ and variance is $\text{Var}\left(\delta_{i}\right)=\frac{{\left(1-a\right)}^{2}}{3}$. We remark that a normal distribution was not considered since it is defined for negative numbers, while rates are positive. Figure 5 shows the critical point estimations in this scenario. The same conclusions we obtained for Fig. 1 also apply here. The same trend is observed, in which the critical point moves to the left. The main observed difference is that, in this case, the critical predictions are poorer when the variance is larger. Although the error is larger in these cases, our predictions are still better than the standard QMF formulation.

## APPENDIX D: SUPERCRITICAL BEHAVIOR

Aside from the critical behavior, discussed in the main text, it is also instructive to evaluate the supercritical behavior of our dynamics. Figure 6 shows the phase diagram for Erdős-Rényi and PL networks with γ=2.1,2.7,3.5. It is clear that not only the critical but also the supercritical behavior changes for different values of α. Note that, in some cases, the lower-α regime is similar to the higher-α regime of a different structure. This is particularly clear for the PL case of γ=3.5 and α=2.0, which is very similar to γ=2.7 and α→∞. A similar effect is also visible for other parameters. This is a particularly important issue if one uses the dynamical process to infer the network structure [44]. Furthermore, for sufficiently large values of λ all the curves tend to converge; however, this convergence is arguably slower for PL networks, as emphasized in the inset of Fig. 6.

## APPENDIX E: CONTACT PROCESS

In the continuous formulation, the CP is modeled as a set of Poisson processes, where each edge has the spreading rate defined as $\lambda_{ij}=\frac{\lambda }{k_{i}}$. The recovery rate is kept fixed for all nodes. In this dynamical process, the average number of contacts of each node is the same for all individuals in the network regardless of their degree. This process is described by the probability transition matrix P, hence $\tau_{c}^{\mathrm{QMF},\mathrm{CP}}=1$. In Fig. 7 we can observe that the critical point predictions are not as accurate as for the previous case we studied in the main text [Fig. 3(a)]. This was anticipated in [46,47]. Interestingly, a better prediction for the critical point in the CP is obtained using the heterogeneous mean field, which predicts $\tau_{c}^{\text{HMF,}\;\text{CP}}=\frac{\langle k\rangle }{\langle k\rangle -1}$ [47]. Thus, the mismatch between prediction and estimated critical points, in both cases, seems to be related to dynamical correlations.

Due to its similarities, it is instructive to compare this model with our dynamic-structure correlated model, i.e., $\delta_{i}=k_{i}$. In Fig. 7 we show the finite-size analysis for the CP case on top of power-law networks with exponents γ=2.1,2.7,3.5. We remark that this process has a finite critical point. Thus, comparing with Fig. 3(a), we observe that both processes present a similar behavior as a function on N. However, note that both models have slightly different estimated critical points. Aside from that, our results also suggest that the convergence to the asymptotic value seems to be faster in the CP case. We can thus conclude that although both processes have the same predictions for the critical point, the second-order effects are different in both of them.

## APPENDIX F: LOCALIZATION PROPERTIES

On top of some structures and just above the critical point, the mean-field theory predicts that the disease can be restricted (localized) in a subgraph [3,48]. This phenomenon is known as metastable localization. In the standard scenario, this property depends only on the structure, more specifically, on the leading eigenvector of A. This property is very robust with respect to the spreading mechanism. In [48] the authors showed that bursts of infection are not sufficient to turn a localized spreading into a delocalized spreading. Interestingly, in our model, we can show that the recovery rates are enough to alter the localization. We remark that the control of the localization of diseases is still an open and interesting problem, as also pointed out in [48].

As anticipated, our model plays an important role in the localization of the leading eigenvector. More specifically, the matrix Q might present a different localization pattern if compared to the adjacency matrix. To quantify localization we use the so-called inverse participation ratio, initially used in epidemics in [3]. Formally, it is given as

(F1) $$\mathrm{IPR}=\sum_{i}^{N}v_{i}^{4},$$

where v is the normalized leading eigenvector, i.e., ∥v∥=1. As shown in [37] and recently formalized in [48], the eigenvector is localized in a subextensive portion, i.e., $\mathrm{IPR}∼O(N^{-\nu })$ with 0<ν<1. Thus, in the fully delocalized case, $\mathrm{IPR}∼O(N^{-1})$, and in the fully localized case, IPR∼O(1). As expected, Fig. 8 shows that in an ER network the IPR scales with ν≈1, ν<1 for a PL network with γ=2.1 [Fig. 8(a)], and a PL network with γ=3.5 is localized [Fig. 8(b)]. Complementarily, when $\delta_{i}=k_{i}$, the leading eigenvector of P=Q is homogeneously distributed; therefore $\mathrm{IPR}∼O(N^{-1})$ and the disease is fully delocalized, which suggests that this is an effective strategy for full delocalization.

Furthermore, Fig. 8 also shows that when heterogeneous recovery rates are considered, the IPR might also change. It is possible that a network that exhibits full delocalization in the standard case (light pink circles for an ER network in Fig. 8) will instead exhibit localization when the recovery rates and structure are correlated (blue triangles in Fig. 8). Thus, by introducing localization and/or tuning, its scaling exponent ν is not trivial. More specifically, one might naively use the recovery rates as $\delta_{i}=\frac{k_{i}}{k_{\text{PL}}}$ as a strategy. In this case, $k_{i}$ would delocalize the matrix, while $k_{\text{PL}}$ would control the new IPR towards the distribution $k_{\text{PL}}$. To evaluate this strategy, we fixed the structure to an ER network. Next we defined $k_{\text{PL}}$ as a PL degree distribution and opted for a discrete approach for $k_{\text{PL}}$, which allowed for a comparison with the localization properties of a network with the same degree distribution, i.e., $k_{\text{PL}}$. However, as we observed in Fig. 8, this was insufficient to control localization. In the evaluated cases, the resulting matrix Q is localized. Note that $k_{\text{PL}}∼k^{-2.1}$ is partially delocalized [dark pink squares in Fig. 8(a)], but its composition with the delocalized structure results in a localized IPR. The case of $k_{\text{PL}}∼k^{-3.5}$ is less surprising, but also emphasizes the role of heterogeneity in the recovery times in disease localization. Therefore, with this simple strategy, we could not control the localization for an arbitrary ν.
